# Flavobacterium psychraquaticum sp. nov., isolated from water system of Atlantic salmon (Salmo salar) smolts cultured in Chile

**DOI:** 10.1099/ijsem.0.006309

**Published:** 2024-04-02

**Authors:** Ruben Avendaño-Herrera, Mónica Saldarriga-Córdoba, Matías Poblete-Morales, Rute Irgang

**Affiliations:** 1 Universidad Andrés Bello, Laboratorio de Patología de Organismos Acuáticos y Biotecnología Acuícola, Facultad de Ciencias de la Vida, Viña del Mar, Chile; 2 Interdisciplinary Center for Aquaculture Research (INCAR), Viña del Mar, Chile; 3 Centro de Investigación Marina Quintay (CIMARQ), Universidad Andrés Bello, Quintay, Chile; 4 Centro de Investigación en Recursos Naturales y Sustentabilidad, Universidad Bernardo O'Higgins, Santiago, Chile

**Keywords:** Atlantic salmon, aquatic, Chile, *Flavobacterium*, smolts

## Abstract

Strain LB-N7^T^, a novel Gram-negative, orange, translucent, gliding, rod-shaped bacterium, was isolated from water samples collected from an open system of Atlantic salmon (*Salmo salar*) smolts in a fish farm in Chile during a flavobacterial infection outbreak in 2015. Phylogenetic analysis based on 16S rRNA sequences (1337 bp) revealed that strain LB-N7^T^ belongs to the genus *Flavobacterium* and is closely related to the type strains *Flavobacterium ardleyense* A2-1^T^ (98.8 %) and *Flavobacterium cucumis* R2A45-3^T^ (96.75 %). The genome size of strain LB-N7^T^ was 2.93 Mb with a DNA G+C content 32.6 mol%. Genome comparisons grouped strain LB-N7^T^ with *Flavobacterium cheniae* NJ-26^T^, *Flavobacterium odoriferum* HXWNR29^T^, *Flavobacterium lacisediminis* TH16-21^T^ and *Flavobacterium celericrescens* TWA-26^T^. The calculated digital DNA–DNA hybridization values between strain LB-N7^T^ and the closest related *Flavobacterium* strains were 23.3 % and the average nucleotide identity values ranged from 71.52 to 79.39 %. Menaquinone MK-6 was the predominant respiratory quinone, followed by MK-7. The major fatty acids were iso-C_15 : 0_ and anteiso-C_15 : 0_. The primary polar lipids detected included nine unidentified lipids, two amounts of aminopospholipid and phospholipids, and a smaller amount of aminolipid. Phenotypic, genomic, and chemotaxonomic data suggest that strain LB-N7^T^ (=CECT 30406^T^=RGM 3221^T^) represents as a novel bacterial species, for which the name *Flavobacterium psychraquaticum* sp. nov. is proposed.

## Introduction

The genus *Flavobacterium* was first proposed by Bergey *et al.* [1], and its description was revised by Bernardet and Bowman in 2015 [2]. Members of the genus form different, yellow-coloured colonies on the agar plates on which they grow. The different species are found in warm to polar environments and have been described from plants, soils, and in a variety of animals (e.g., birds, mammals, and fish), in freshwater and marine environments [3]. At the time of writing, this genus comprises 297 species with valid published names (https://lpsn.dsmz.de/genus/flavobacterium; accessed 9 August 2022). Multiple bacterial species within the family *Flavobacteriaceae* species are considered pathogenic for numerous cultured fish species throughout the world, including *Flavobacterium psychrophilum*, *Flavobacterium columnare*, and *Flavobacterium branchiophilum* [4].

Chile is currently ranks as the second-largest producer of farmed salmon worldwide. Since its initial description in 1995 [5], *F. psychrophilum* has been a major contributor to losses during the freshwater culturing stage in salmonid fish farms [6]. In addition, several other members of the genus *Flavobacterium* have been implicated in outbreaks and linked to fish mortalities, including new discovered species. Notably, some flavobacterial outbreaks have been attributed to *F. columnare* [7]. The novel species *Flavobacterium araucananum* and *Flavobacterium salmonis* were isolated from diseased Atlantic salmon (*Salmo salar*) [8,9], and *Flavobacterium chilense* was identified in diseased rainbow trout (*Oncorhynchus mykiss*) [9]. In this study, we present the isolation and characterization of strain LB-N7^T^, a member of the genus *Flavobacterium*, recovered from freshwater culture systems containing Atlantic salmon smolts in May 2015.

## Isolation and ecology

To ascertain the aquatic microbiota in the freshwater culture systems of Atlantic salmon smolts at a fish farm in the Araucanía Region, Chile (38 ° 51′ 11.657″ S 71 ° 41′ 42.05″ W), bacterial isolation procedures were performed. This process entailed collecting 50 ml water samples, followed by serial dilution in an 0.85 % sterile saline solution and subsequent 100 µl of each dilution spreading on tryptone yeast extract salts (TYES) agar plates, as described by Valdebenito and Avendaño-Herrera [10]. The aim of the sampling was to assess the bacterial load and determine whether the yellow-orange or pigmented colonies were attributable to *F. psychrophilum*, given that the farmed fish were experiencing an infectious disease caused by this micro-organism.

All plates were incubated aerobically at 18^ ^°C for 1 week and examined daily for growth detection. A mixed bacterial culture was obtained, dominated by white and non-pigmented colonies, except for the presence of the orange-coloured LB-N7^T^ strain with the lowest number of colony-forming units (5×10^5^ c.f.u. ml^−1^). When these colonies were subjected to PCR, specific for detecting *F. psychrophilum* [11], the expected 1088 bp amplification product was not observed. In contrast, the control DNA from the type strain NCIMB 1947^T^ was positively identified as belonging to *F. psychrophilum*. These colonies were examined by phase-contrast microscopy, and cells showing a unique morphology were purified. A single colony representative of the strain LB-N7^T^ was selected and stored in TYES broth supplemented with 10 % glycerol (v/v) and Criobille tubes (AES Laboratoire) at −80 °C until use.

All experiments and animal handling were conducted in compliance with CONICYT’s ethical standards and approved by the Ethics Committee of Universidad Andrés Bello (Authorization No. 015/2015).

## 16s rRNA gene phylogeny

For the taxonomic identification of strain LB-N7^T^, colonies from pure culture growth were selected, and genomic DNA was extracted using the InstaGene Matrix (Bio-Rad) following the manufacturer’s instructions. The 16S rRNA was amplified using the universal primer pair pA (3′-AGAGTTTGATCCTGGTCAG-5′) and pH (3′-AAGGAGGTGATCCAGCCGCA-5′) [12]. The PCR reaction was performed using GoTaq Flexi G2 (Promega) in a final volume of 50 µl, that contained 10 ng of DNA template, 5×GoTaq Flexi Buffer (Promega), 100 mM MgCl_2_, 10 mM dNTPs, and 10 µM of each primer. The PCR reaction was performed with the GeneTouch thermal cycler TC-EA (Hangzhou Bioer Technology Co., Ltd.) using the following settings: 94 °C for 5 min; followed by 35 cycles at 95 °C for 1 min, 61 °C for 1 min, and 72 °C for 2 min; with a final extension at 72 °C for 10 min. The amplification product was then sequenced for Sanger capillary electrophoresis sequencing by Macrogen Inc. (Seoul, Republic of Korea).

The 16S rRNA sequence obtained was edited using Geneious Prime version 2023.0.4 (www.geneious.com), with manual verification performed in BioEdit version 7.2.5 [13]. Pairwise comparison of the 16S rRNA gene sequence with the most closely related type strains were conducted using blast against the 16S rRNA gene-sequence database in EzBioCloud [14]. Phylogenetic trees for the 16S rRNA sequences, including the 25 closest type strains of the genus *Flavobacterium*, were reconstructed using the neighbour-joining method in mega version 11.011 [15] and Bayesian inference in MrBayes version 3.0B4 [16]. mega was used to estimate the best-fitting model for the nucleotide evolution. For Bayesian inference analysis, the GTR+G+I evolution model was employed as the best fit, as determined by the Bayesian information criterion [17].

**Fig. 1.**
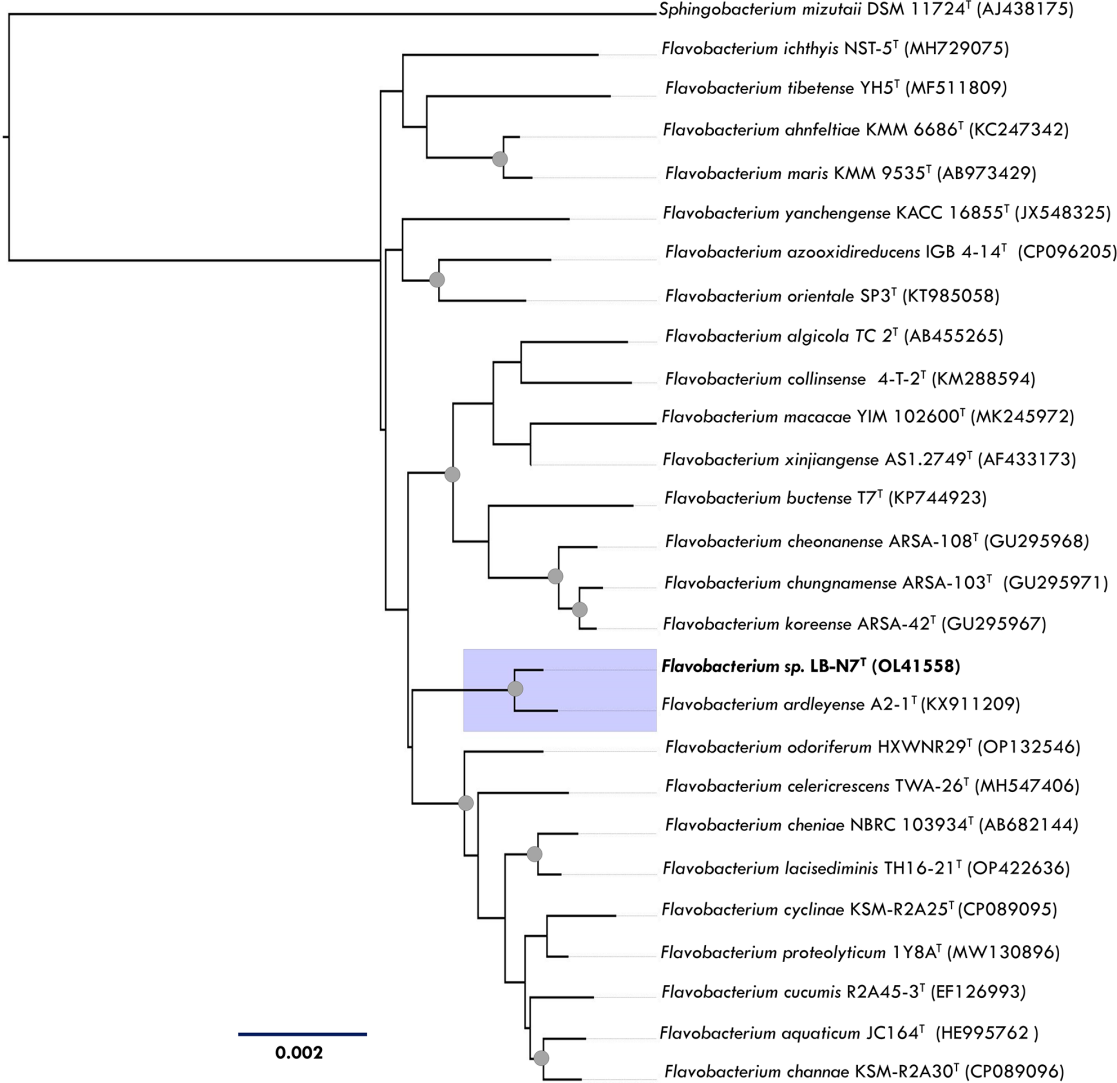
The taxonomic position of strain LB-N7^T^ within the 25 type species of the genus *Flavobacterium* inferred in the mega X program by the neighbour-joining algorithm using 1337 bp of the 16S rRNA gene. Evolutionary distances were computed using the *p* distance, with bootstrap support of 10 000 replicates. Nodes with bootstrap support ≥80 % are indicated in each node. *Sphingobacterium mizutaii* DSM 11724^T^ (AJ438175) was used as an outgroup.

The 16S rRNA gene sequence of strain LB-N7^T^ (accession number OL415581) is 1337 bp in length. It exhibited the highest similarity of 98.8 % to *Flavobacterium ardleyense* A2-1^T^ (KX911209), followed by a 96.75 % similarity to *Flavobacterium cucumis* R2A45-3^T^ (EF126993). Phylogenetic analysis using the neighbour-joining algorithm revealed that strain LB-N7^T^ is most closely related to *F. ardleyense* A2-1^T^, forming a well-supported clade (99 %), as shown in Fig. 1. Similarly, Bayesian inference (posterior probability=0.98) positioned strain LB-N7^T^ as a sister group to *F. ardleyense* A2-1^T^, as depicted in (Fig. S1, available in the online version of this article). These phylogenetic trees collectively affirm the placement of strain LB-N7^T^ within the genus *Flavobacterium* cluster.

## Genome analysis, phylogeny and features

Whole genome sequencing of strain LB-N7^T^ was conducted by the Fraunhofer Chile Research Foundation. The NextEra XT library preparation kit was used to prepare the libraries, and sequencing was performed on a MiSeq instrument using 2×300 cycles of paired end v3 chemistry (Illumina Inc.). The process yielded 469 471 read pairs, which were assembled using SPAdes version 3.11.136 software [18]. The assembly quality was evaluated with quast version 5.1 [19].

The draft genome has a total size of 2 936 389 bp, consisting of 30 contigs (>1000 bp). It has an N50 value of 218 502 bp, with the shortest contig being 1337 bp and the longest at 882 882 bp. The genome’s G+C content is 32.6 mol%.

To test whether strain LB-N7^T^ is closely related to other previously described species, its genome sequence data were upload to the Type (Strain) Genome Server (https://tygs.dsmz.de/) for comprehensive whole-genome-based taxonomic analysis [20]. This analysis identified 15 closely related *Flavobacterium* type strains. Pairwise comparisons of LB-N7^T^ genome with these type strains revealed digital DNA–DNA hybridization (dDDH) values below the species delineation threshold of 70 % [21], suggesting that LB-N7^T^ represents a new species. The highest dDDH value observed was 23.3 %, recorded with *Flavobacterium odoriferum* HXWNR29^T^ (GCA_025499515) [22], and 22.6 % with *Flavobacterium celericrescens* TWA-26^T^ (GCA_011392075) [23], *Flavobacterium cheniae* DSM 22462^T^ (GCA_004363695) [24] and *Flavobacterium lacisediminis* TH16-21^T^ (GCA_025909975) [25].

**Fig. 2.**
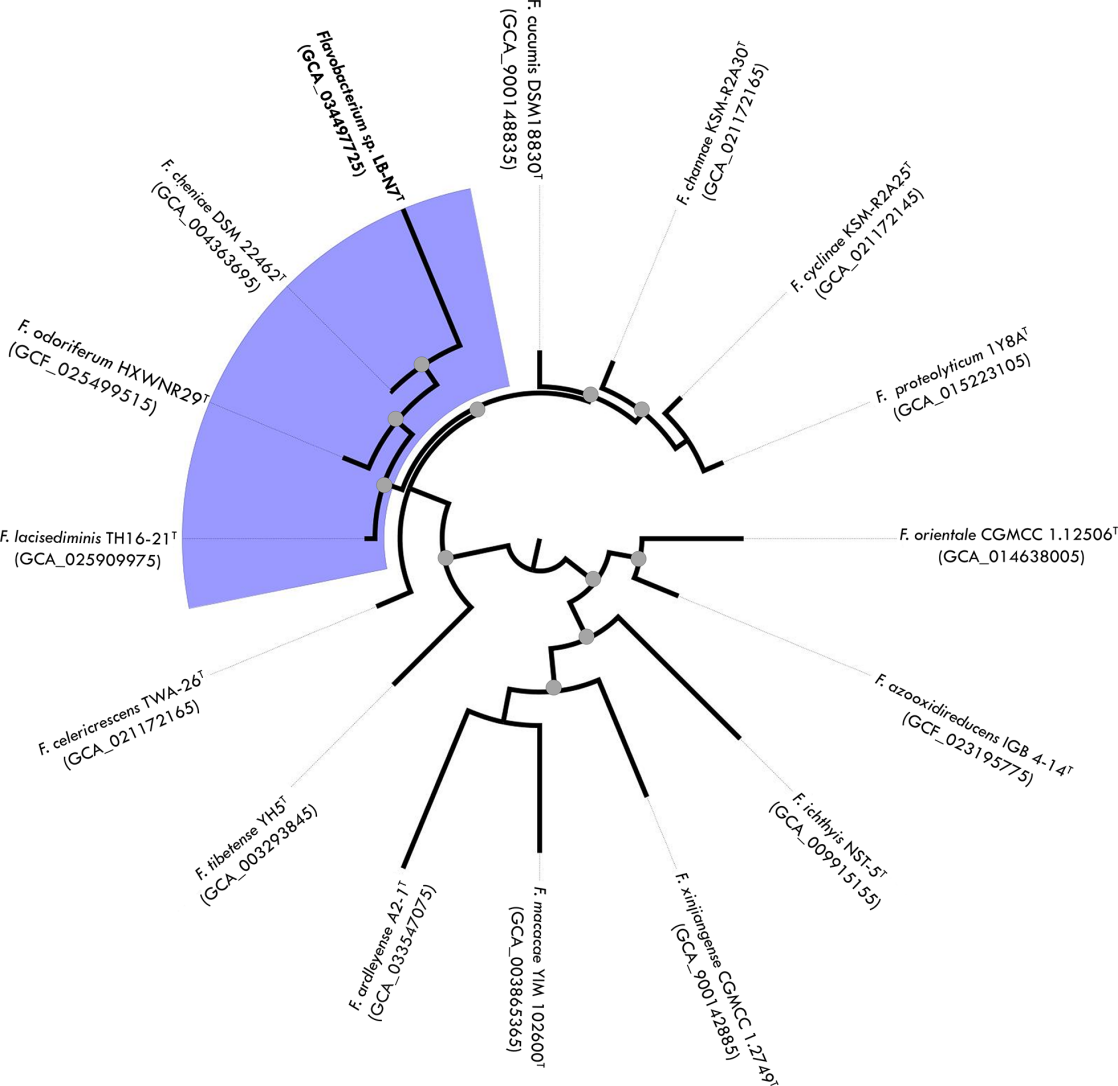
Phylogenetic relationship among strain LB-N7^T^ and 15 closely related *Flavobacterium* type strains. Phylogeny was performed with whole-genome sequence data in the Reference Sequence Alignment-Based Phylogeny builder inferred via PhyML. Grey dots indicate bootstrap support >80 %.

These 15 genome sequences were obtained from NCBI database for phylogenetic analysis. The phylogenetic relationships between LB-N7^T^ and 15 type strains with available whole genome were determined using realphy (Reference sequence Alignment-based Phylogeny builder inferred via PhyML [26]) (Fig. 2). The phylogenetic tree positioned LB-N7^T^ in a well-supported group (boostrap support of 99 %) comprising *F. cheniae* DSM 22462^T^ (GCA_004363695), *Flavobacterium odoriferum* HXWNR29^T^ (GCA_025499515) and *F. lacisediminis* TH16-21^T^ (GCA_025909975).

The genome was further analysed in comparison with the available genome of the *Flavobacterium* type strain, employing pairwise average nucleotide identity (ANI) calculations on the JSpecies web server [27]. The following parameters were used to establish a significantly different species: <94 % identity for blast analysis (ANIb), <96 % MUMer analysis (ANIm), and <0.99 for tetranucleotides frequency analysis (TETRA) [28]. The values derived from the analyses comparing strain LB-N7^T^ with other *Flavobacterium* type strains fell below the cutoff for species delineation, which is 95~96 %. These values ranged from 69.61 to 80.09 % for ANIb, and from 83.12 to 84.37 % for ANIm. For TETRA, the values were between 0.82 and 0.94. The highest values observed were 80.09 % for ANIb with *F. odoriferum* HXWNR29^T^, 84.37 % for ANIm with * F. lacisediminis* TH16-21^T^; and 0.94 for TETRA with *F. odoriferum* HXWNR29^T^.

The LB-N7^T^ genome was annotated in the Bacterial and Viral Bioinformatics Resource Center (www.bv-brc.org/app/Annotation). Whole genome annotation yielded 2835 coding sequences, of which 1361 were protein-encoding genes without functional assignment, and 41 tRNAs. Genes involved in secretion systems were identified using TXSScan 2.0+galaxy2 (https://galaxycat.france-bioinformatique.fr/tools/8579). *In silico* genome analysis revealed genes related to secretion system such as T1SS and T9SS. Iron-related protein families were searched using FeGenie software version 1.2 [29]. Families of iron-related protein involved in hemin transport, iron transport, siderophore transport, transcriptional regulation, and iron storage were identified. Haemolysins and related proteins containing CBS domains were also found, as detailed in Table S1.

The search for resistance genes was conducted using the Resistance Gene Identifier within the Comprehensive Antibiotic Resistance Database [30]. This search identified two genes associated with antibiotic resistance, adhering to strict criteria: erythromycin esterase (EreD family, a macrolide antibiotic, with best identities of 98.53 %) and tetracycline inactivating enzyme (Tet X, a tetracycline antibiotic, with best identities of 99.48 %).

Additionally, putative virulence factors were investigated in the Virulence Factor Database (www.mgc.ac.cn/VFs/ [31]). Within this database, various categories of virulence factors were identified, including adherence, antiphagocytosis, iron uptake, secretion system, toxins, immune evasion, and stress adaptation (Table S2).

## Physiology and chemotaxonomy

For phenotypic and comparative purposes, strain LB-N7^T^ was routinely cultured on TYES agar plates, aerobically incubated at 25 °C for 48–72 h. Additionally, growth was assessed on various agar media, including Reasoner’s 2A (R2A) agar (Oxoid), blood agar (Oxoid, with 5 % defibrillated sheep blood), brain heart infusion agar (Oxoid), Luria–Bertani agar (Oxoid), 2216 marine agar (BD Difco), Mueller–Hinton agar (Oxoid), nutrient agar (Oxoid), trypticase soya agar (BD Difco), McConkey (Oxoid), thiosulphate–citrate–bile salts–sucrose (TCBS; Oxoid) and TYES. After 2 weeks of incubation, the presence or absence of growth was noted. Notably, robust growth was observed on all media tested, except that no growth was observed on McConkey or TCBS agars. The most significant growth occurred on TYES agar plates (Fig. S2).

Gram staining and cell morphology were analysed using light microscopy at ×1000 magnification with a Motic microscope, identifying Gram-negative rod cells. Cell size, at 0.5 µm×5.0–7.0 µm, was measured with a Zeiss Auriga compact field emission scanning electron microscope (Fig. S3). Gliding motility was evident in 24 h culture in TYES broth. Strain LB-N7^T^ exhibited negative catalase and positive oxidase activities, contrasted with only positive results in closely related species, when tested with 24 h colonies treated with 3 % hydrogen peroxide and an oxidase reagent dropper (BD BBL), respectively. Flexirubin and Congo red pigments presence in fresh LB-N7^T^ colonies were tested according to Bernardet *et al.* [32], yielding positive and negative results for these absorptions. Considering that the culture medium commonly used for the taxonomic identification of members of the genus *Flavobacterium* is R2A medium, some tests were conducted in this medium. Optimal growth temperature for LB-N7^T^ was determined using agar plates incubated at various temperatures: 5, 10, 15, 18, 22, 25, 28, 30 and 37 °C. Notable growth was observed at 5–25 °C, with the optimal range being 15–25 °C. The tolerance of strain LB-N7^T^ to different pH and NaCl concentrations was assessed on R2A agar plates prepared with a pH range of pH 5.0–10.0 (adjusted in one unit interval using 1 N HCl and 1 M NaOH) and NaCl concentrations of 0–6 % (w/v). LB-N7^T^ showed optimal growth with 0–1 % NaCl concentrations, exhibiting weak growth at 2 % NaCl and no growth at 3–6 % NaCl, across pH ranges of pH 6.0–10.0. Hydrolysis tests for casein (1 % w/v), carboxymethyl-cellulose degradation (0.5 %, w/v), DNA (Liofilchem), egg-yolk precipitation (5 % v/v, Liofilchem), gelatin (2 % w/v), l-tyrosine (0.5 % w/v), pectin (0.5 % w/v), starch (0.5 % w/v), as well as Tween 20 and 80 (v/v), were performed on R2A agar plates. The strain showed positive reactions for amylase, gelatinase, and tyrosine hydrolysis but grew on plates prepared with casein, DNAse, and Tween 80, even though no positive hydrolysis was detected. No growth was observed on culture media prepared with carboxymethyl-cellulose, egg yolk, pectin, nor Tween 20.

Enzymatic and substrate reactions using the APIZYM, API20E and API20NE systems were conducted according to the manufacturer's instructions, except that for comparative purposes, the API ZYM strips were incubated for 48 and 72 h. For the API50CH kit, the API50CHB/E medium recommended for Gram-negative bacteria/*Enterobacteriaceae* was used. Strong reactions were noted with alkaline and acid phosphatase, leucine arylamidase and trypsin; weak reactions with esterase (C4), esterase lipase (C8) and valine arylamidase. No reactions were observed for substrates including lipase (C14), cystine arylamidase, a-chymotrypsin, a- and b-galactosidase, b-glucuronidase, a- and b-glucosidase, N-acetyl-b-glucosaminidase, a-mannosidase and a-fucosidase (API ZYM). The API 20E system indicated arginine dihydrolase activity and weak gelatin hydrolysis, while API 20NE showed hydrolysis of gelatin; and reduction of nitrate and nitrite were negative. With API 50CH revealed fermentation of d- and l-arabinose, d-ribose, d-xylose, d-galactose, d-glucose, d-fructose, d-mannose, melibiose, trehalose, d- and l-fucose, potassium gluconate, potassium 2-ketogluconate and potassium 5-ketogluconate. Unmentioned reactions remained negative throughout the incubation. The main characteristics distinguishing strain LB-N7^T^ from its closest related species is shown in Table 1.

**Table 1.** Phenotypic results and differentiating features of strain LB-N7^T^ and its relative neighbours Strains: 1, LB-N7^T^; 2, *Flavobacterium cheniae* NJ-26^T^ [24]; 3, *Flavobacterium odoriferum* HXWNR29^T^ [22]; 4, *Flavobacterium lacisediminis* TH16-21^T^ [25]. +, Positive reaction; −, negative reaction or no growth; w, weak reaction; nd, no data.

Characteristic	1	2	3	4
Isolation source	Smolt water system	Sediment	Activated sludge sample	Lake sediment
Colonies	Orange	Yellow	Yellow	Cream-yellow
Catalase/oxidase	−/+	+/+	+/+	+/+
Flexirubin	+	−	−	−
Hydrolysis of:				
Aesculin	w	nd	+	nd
Casein	−	−	+	+
Carboxymethyl-cellulose	−	nd	−	−
Gelatin	w	+	+	+
Starch	+	w	−	w
Tween 20/80	−/−	nd/−	+/−	nd/−
l-Tyrosine	+	+	nd	nd
Temperature range (optimum) for growth (°C)	5–25 (15–25)	18–32 (28)	4–40 (30)	10–35 (20)
Salt tolerance range (optimum) for growth (%)	0–2 (0–1)	0–0.8 (0)	0–1.5 (0)	0–0.5 (0)
pH range (optimum) for growth	6.0–10.0 (7.0–10.0)	6.5–8.0 (7.5)	6.5–9.0 (7.0)	3.5–9.0 (7.0)
API ZYM assay:				
Alkaline phosphatase	+	w	+	nd
Esterase (C4)	w	−	+	nd
Esterase lipase (C8)	w	−	+	nd
Lipase (C14)	−	w	−	nd
Cystine arylamidase	−	−	+	nd
Leucine arilamidase	+	−	+	nd
Valine arilamidase	w	−	+	nd
Acid phosphatase	+	−	+	nd
Trypsin	+	−	+	nd
Naphthol-AS-BI-phosphohydrolase	+	−	+	nd
α- and β-Glucosidase	−, −	−, −	+, −	nd
*N*-Acetyl-β-glucosaminidase	−	−	+	nd
API 20E assay:				
Nitrate reduction	−	+	−	+
API 50CH assay:				
Aesculin	w	nd	+	nd
d- and l-Arabinose	+, +	−, −	−, −	nd, −
d-Fructose	+	−	nd	nd
d- and l-Fucose	+, +	nd	nd	nd
d-Galactose	+	−	nd	+
d-Glucose	+	−	nd	+
d-Mannose	+	−	nd	+
H_2_S production	−	w	−	+
Melibiose	+	−	nd	nd
d-Ribose	+	−	nd	+
d-Xylose	+	−	nd	−
l-Rhamnose	−	−	−	+
Melibiose	+	−	−	nd
Potassium gluconate	+	nd	nd	nd
Trehalose	+	−	nd	−
Sucrose	w	−	−	−
Potassium 2-ketogluconate	+	nd	nd	nd
Potassium 5-ketogluconate	+	ni	ni	ni
Predominant fatty acids (>10 %)	iso-C_15 : 0_ and anteiso-C_15 : 0_	C_15 : 0_, iso-C_15 : 0_ and iso-C_17 : 1_ω9*с*	iso-C_15 : 0_, iso-C_17 : 0_ 3-OH and iso-C_15 : 1_G	iso-C_15:0_, iso-C_16:0_, C_15 : 1_ G and iso-C_16 : 0_ 3-OH
DNA G+C content (mol%)	32.6	40.6	31.8	32.2

The fatty acid composition of strain LB-N7^T^ was analysed from cells cultured on R2A agar plates at 25 °C for 72 h. This analysis was conducted at the Bacterial Section of the Colección Española de Cultivo Tipo (Valencia, Spain), according to the midi Microbial Identification System guidelines [33]. Gas chromatography was performed using an Agilent 6850 system (Agilent Technologies) with a Sherlock midi microbial identification system, applying the TSBA6 protocol [34]. The predominant fatty acids (>10 %) were identified as iso-C_15 : 0_ and anteiso-C_15 : 0_, as shown in Table 2.

**Table 2.** Whole-cell fatty acid composition of LB-N7^T^ and the type strains of related *Flavobacterium* species Strains: 1, LB-N7^T^; 2, *F. cheniae* NJ-26^T^ [24]; 3, *F. odoriferum* HXWNR29^T^ [22]; 4, *F. lacisediminis* TH16-21^T^ [25]. Major components (>10 %) are highlighted in bold. –, not detected or no data; tr, trace composition (<1.0 %).

Fatty acid	1	2	3	4
**Straight chain**				
C_13 : 0_	tr	–	–	–
C_14 : 0_	2.1	–	–	–
C_16 : 0_	tr	2.3	1.1	1.1
C_13 : 1_ at 12–13	tr	–	–	–
**Branched chain**				
iso-C_16 : 1_ h	1.2	–	–	–
iso-C_15 : 1_ G	6.8	**10.9**	**10.8**	**11.3**
iso-C_16 : 1_ G	–	5.1	–	–
iso-C_12 : 0_	tr	–	–	–
iso-C_13 : 0_	tr	–	tr	–
iso-C_14 : 0_	4.0	4.2	–	7.5
iso-C_15 : 0_	**31.0**	**21.7**	**35.0**	**17.8**
iso-C_16 : 0_	2.6	**16.9**	2.2	**20.8**
anteiso-C_13 : 0_	tr	–	–	–
anteiso-C_15 : 0_	**12.1**	2.4	3.3	4.5
anteiso-C_15 : 1_ A	–	–	–	1.7
**Unsaturated**				
C_15 : 1_ ω6*с*	5.0	–	tr	–
C_17 : 1_ ω6*с*	–	–	–	–
C_17 : 1_ ω8*с*	–	–	–	–
**Hydroxy**				
C_15 : 0_ 2-OH	tr	–	–	–
C_15 : 0_ 3-OH	1.7	–	2.0	–
C_16 : 0_ 3-OH	2.9	3.5	1.7	–
C_17 : 0_ 2-OH	1.0	–	–	–
iso-C_14 : 0_ 3-OH	–	1.7	1.1	2.9
iso-C_15 : 0_ 3-OH	7.6	6.3	9.0	5.4
iso-C_16 : 0_ 3-OH	6.5	**10.5**	3.0	**12.4**
iso-C_17 : 0_ 3-OH	5.6	4.9	**11.7**	4.6
Summed features*				
3	4.0	2.8	3.7	–
9	1.3	1.3	5.3	1.9

*Summed feature 3 comprised C_16 : 1_ *ω*7*c* and/or C_16 : 1_ *ω*6*c* and summed feature 9 comprised iso-C_17 : 1_ *ω*9*c* and/or C_16 : 0_ 10-methyl.

**Fig. 3.**
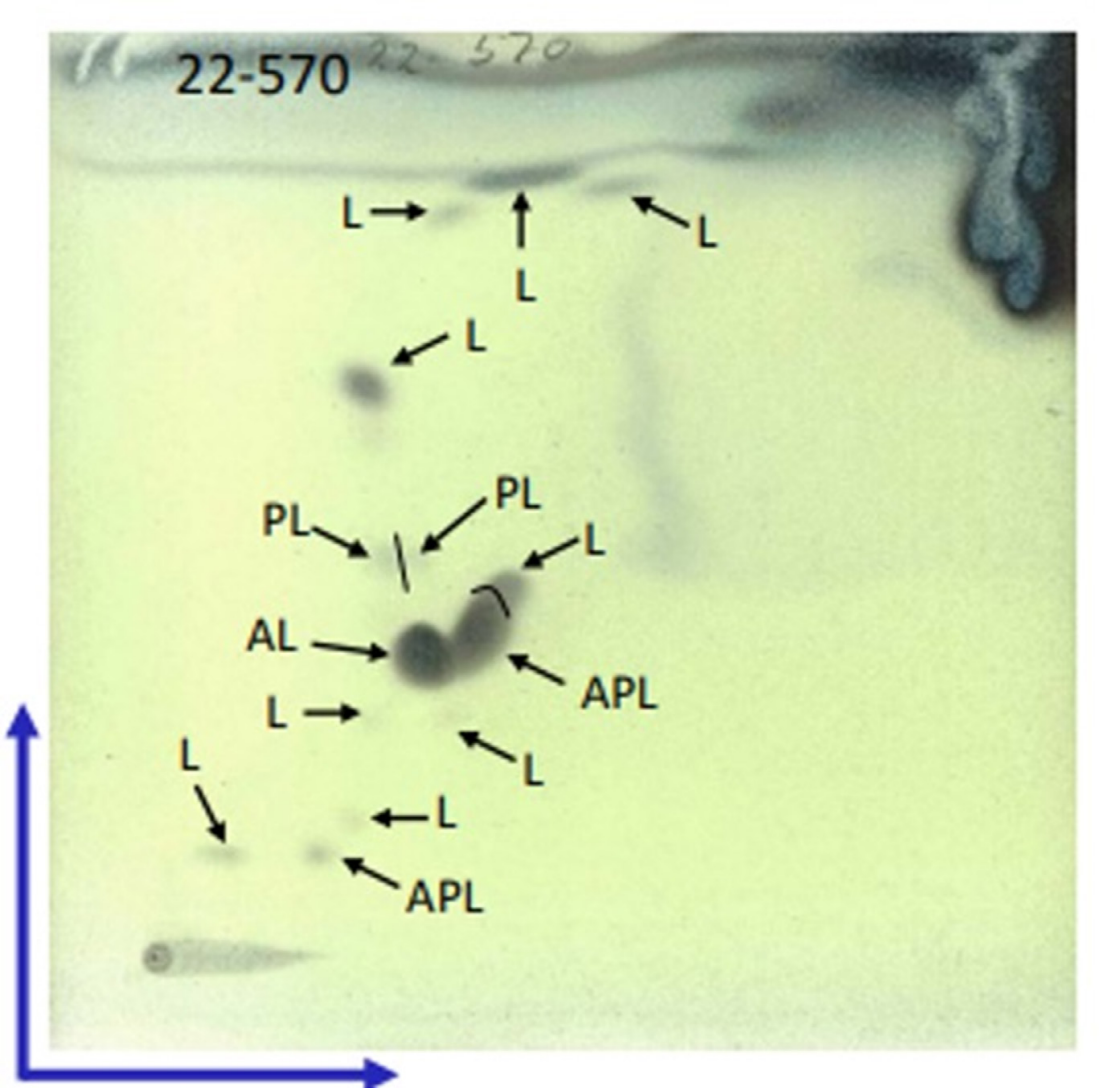
Two-dimensional silica gel thin-layer chromatogram showing the total polar lipids of strain LB-N7^T^. Total lipid material was detected using molybdatophosphoric acid and specific functional groups detected using spray reagent specific for defined functional groups. APL, aminophospholipid (*n*=2); AL, aminolipid (*n*=1); PL, phospholipid (*n*=2) and L, lipids (*n*=9).

Polar lipid analysis and respiratory menaquinone determination were conducted on cells grown on R2A agar plates for 48–72 h at 25 °C. Cells were harvested and resuspended in an isopropanol–water solution (1 : 1, v/v) for analysis at DSMZ Services (Leibinz – Institut DSMZ - Deutsche Sammlung von Mikroorganismen und Zellkulturn GmbH, Braunschweig, Germany), following methods outlined by Bligh and Dyer [35] and Tindall *et al.* [36]. The analysis revealed primarily nine unidentified lipids, equal amounts (*n*=2) of aminophospholipid and phospholipids, and a smaller proportion of aminolipid (Fig. 3). Menaquinone MK-6 was the primary respiratory quinone, followed by MK-7.

Based on the presented results for phylogenomic, genome, biochemical, and physiological analyses, strain LB-N7^T^ appears to be a novel species of the genus *Flavobacterium*, for which the name *Flavobacterium psychraquaticum* sp. nov. is proposed.

## Description of *Flavobacterium psychraquaticum* sp. nov.

*Flavobacteriumpsychraquaticum* (psychr.a.qua'ti.cum. Gr. masc. adj. *psychros*, cold; L. masc. adj. *aquaticus*, found in the water, aquatic; N.L. neut. adj. *psychraquaticum*, psychrophilic and aquatic).

Cells are Gram-stain-negative, exhibit gliding motility, are non-spore-forming long rods, and 0.5×5.0–7.0 µm. Growth occurs on blood agar, brain hearth infusion agar, Luria–Bertani agar, marine agar, Mueller–Hinton agar, nutrient agar, trypticase soy agar and broth, but not on McConkey agar or TCBS. Colonies are orange, translucent, with entire margins, and 2 mm in diameter on R2A agar plates after 72 h of incubation at 25 °C. Growth occurs at 5–25 °C (optimum, 15–25 °C), at pH 6.0–10.0 (optimum, pH 7–10) and with 0–2 % NaCl (optimum, 0–1 %). Flexirubin-type pigments are present, but the Congo red test is negative. Catalase activity is negative and oxidase activity positive. In plate assays, the stain grew on all the different preparations but only gave positive results for amylase, gelatinase, and tyrosine hydrolysis and negative results for casein, carboxymethyl-cellulose degradation, DNA, egg-yolk precipitation, pectin, and Tween 20 and 80. Strong reactions were noted with alkaline and acid phosphatase, leucine arylamidase and trypsin; weak reactions with esterase (C4), esterase lipase (C8) and valine arylamidase. No reactions were observed for substrates including lipase (C14), cystine arylamidase, a-chymotrypsin, a- and b-galactosidase, b-glucuronidase, a- and b-glucosidase, N-acetyl-b-glucosaminidase, a-mannosidase and a-fucosidase (API ZYM). The API 20E system indicated arginine dihydrolase activity and weak gelatin hydrolysis, while API 20NE only showed gelatin hydrolysis. Reduction of nitrate and nitrite were negative. API 50CH revealed fermentation of d- and l-arabinose, d-ribose, d-xylose, d-galactose, d-glucose, d-fructose, d-mannose, melibiose, trehalose, d- and l-fucose, potassium gluconate, potassium 2-ketogluconate and potassium 5-ketogluconate. Reactions not mentioned remained negative throughout the incubation period. The major cellular fatty acids (>10 %) are iso-C_15 : 0_ and anteiso-C_15 : 0._ Four types of polar lipids are detected: nine unidentified lipids, two aminophospholipids, two phospholipids and one aminolipids. Two respiratory quinones are present, MK-6 and MK-7. The genome G+C content of the genome of strain LB-N7^T^ is 32.6 mol%.

The type strain, LB-N7^T^ (=CECT 30406^T^=RGM 3221^T^), was isolated during water sampling in 2015 from the water system of Atlantic salmon smolts reared at a fish farm in Chile. The GenBank accession number for the 16SrRNA gene sequence is OL415581, and the whole genome shotgun project has been deposited at DDBJ/ENA/GenBank under the accession number JAXKVR000000000. The version described in this paper is version JAXKVR010000000. BioProject ID PRJNA1048623, BioSample accession number SAMN38650132 and SRA accession number SRR27238119.

## supplementary material

10.1099/ijsem.0.006309 Uncited Supplementary Material 1.

## Abbreviations

MK: menaquinone
PCR: Polimerase chain reaction
T1SS: type I secretion systems
T9SS: type IX secretion systems
TYES: tryptone yeast extract salts
