# Supplementary material for: Flavobacterium psychraquaticum sp. nov., isolated from water system of Atlantic salmon (Salmo salar) smolts cultured in Chile
Source: Int J Syst Evol Microbiol. 2024 Apr 2;74(4):006309. doi: 10.1099/ijsem.0.006309 (PMC11092233; doi:10.1099/ijsem.0.006309)
Supplement: Uncited Supplementary Material 1. [file ijsem-74-06309-s001.pdf]

Figure S1 The taxonomic position of *Flavobacterium sp*. LB-N7T within the 25 type species of the genus *Flavobacterium* inferred in the MrBayes 3.2.7 program by the Bayesian inference algorithm using 1,337 bp of the 16S rRNA gene. The evolutionary model used was GTR+G+I. Nodes with a posterior probability ≥0.95 are indicated in each node. *Sphingobacterium mizutaii* DSM 11724T (AJ438175) was used as an external group.

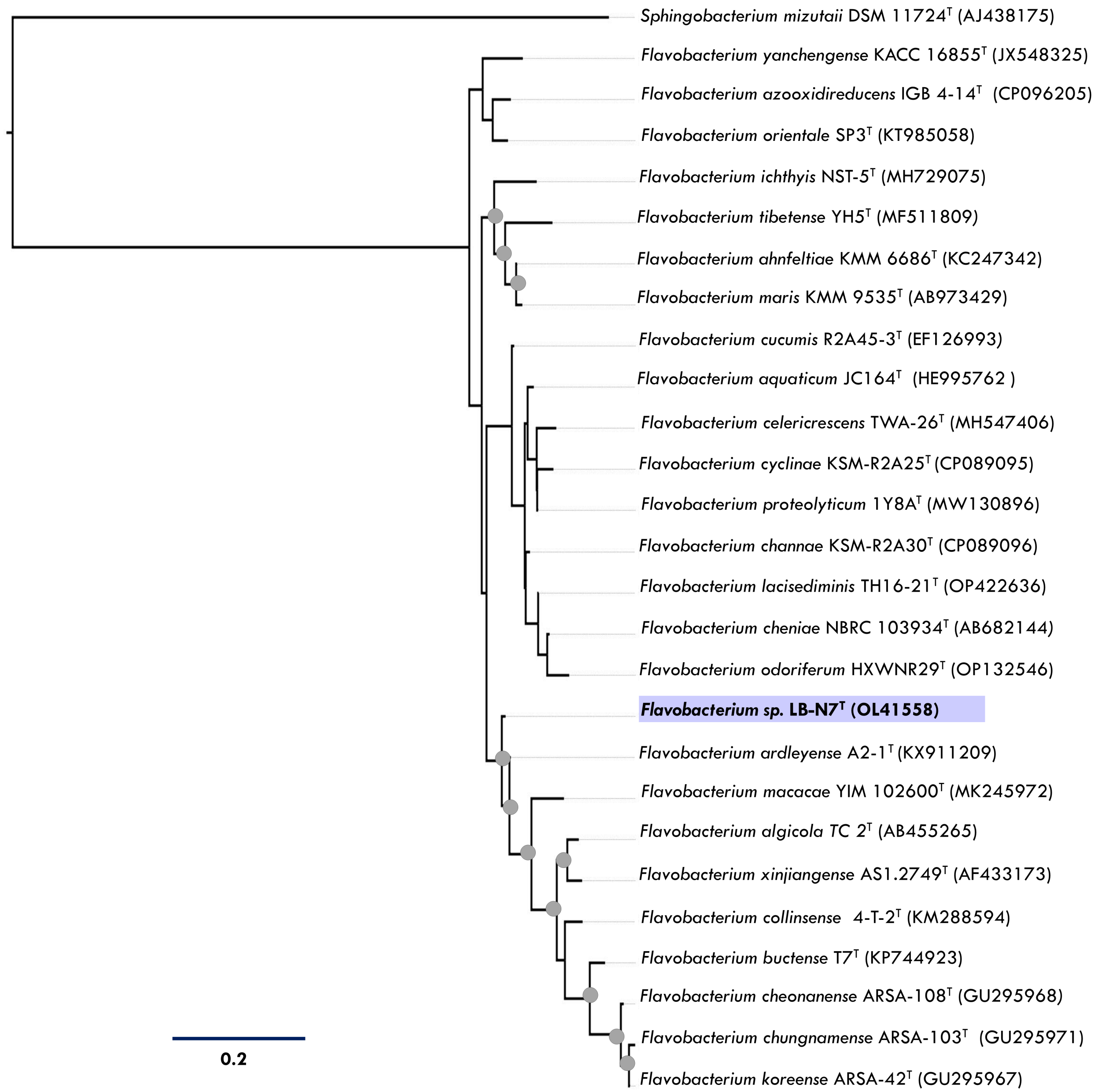

Figure Supplementary 2. Colonies of *Flavobacterium psychroaquaticum* LB-N7^{T} cultured on R2A (left) and TYES (right) agar plates, incubated at 25ºC for 72 h.

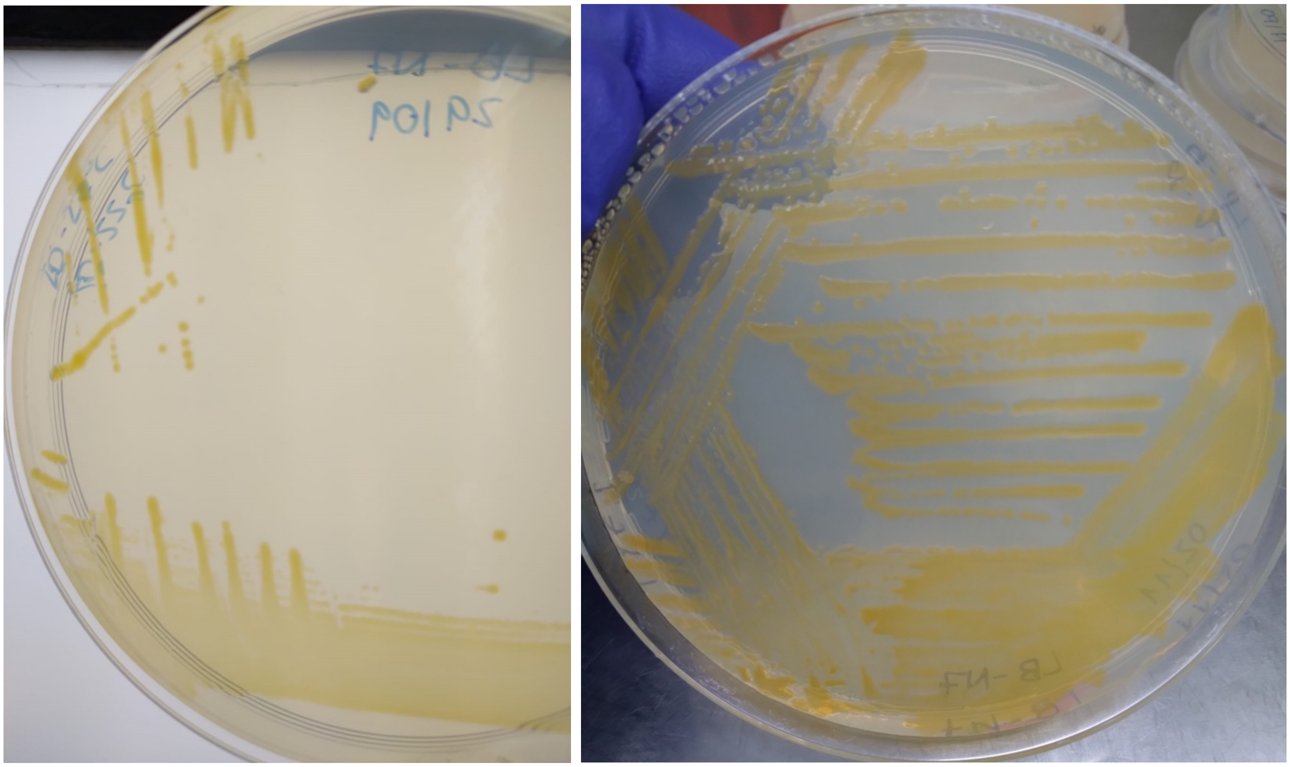

Figure Supplementary 3. Compact field emission scanning electron microscope of strain LB-N7^T grown in TYES broth, incubated at 25ºC for 48 h. Bar 2 µm.

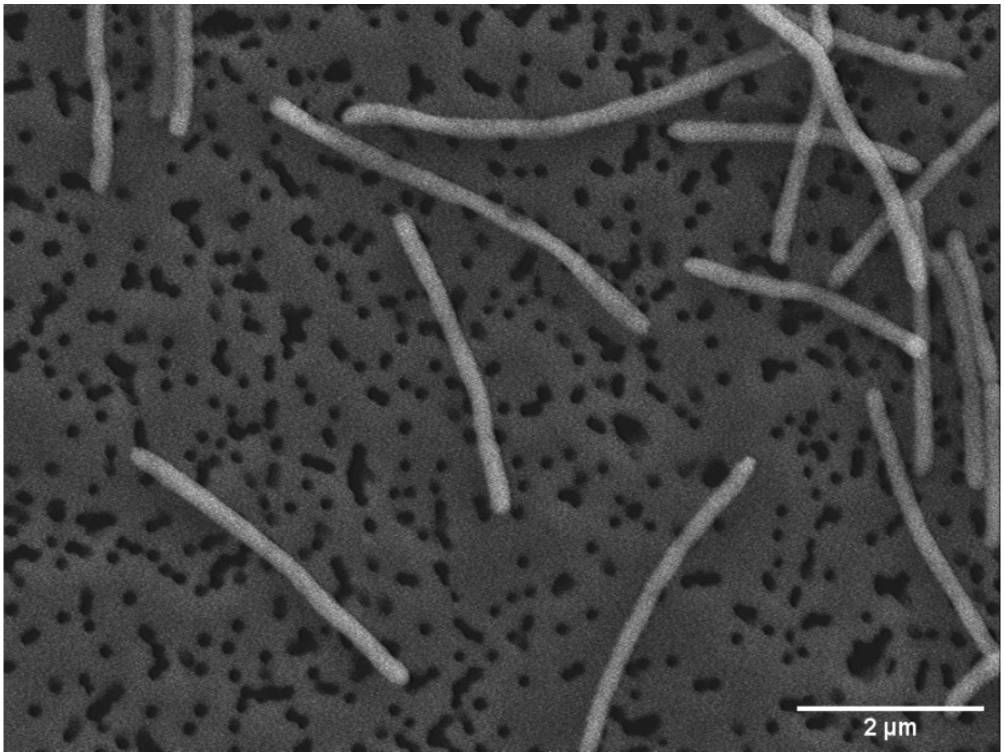

Table S1. Summary of CDS sequences in the strain LB-N7^T related with secretion system and iron related protein families.

Type	Function	Hit	Accession number
Secretion system T1SS	ABC transporter ATP-binding protein	*F. cucumis*	WP_073584418
Secretion system T1SS	ATP-binding cassette domain-containing protein	*F. aquaticum*	WP_111566076
Secretion system T1SS	HlyD family efflux transporter periplasmic adaptor subunit	*F. saliperosum*	WP_023575658
Secretion system T1SS	HlyD family secretion protein	*F. jejuense*	WP_140959288
Secretion system T1SS	efflux RND transporter periplasmic adaptor subunit	*F. bernardetii*	WP_166125799
Secretion system T1SS	Outer membrane protein TolC	*F. celericrescens*	WP_166237149
Secretion system T9SS	gliding motility lipoprotein GldK	*F. channae*	WP_231835188
Secretion system T9SS	gliding motility-associated protein GldL	*F. cucumis*	WP_073580622
Secretion system T9SS	gliding motility-associated protein GldM	*F. cheniae*	WP_133608102
Secretion system T9SS	gliding motility associated protien GldN	*F. celericrescens*	WP_166236529
Secretion system T9SS	gliding motility lipoprotein GldJ	*F. celericrescens*	WP_166237516
Secretion system T9SS	gliding motility-associated protein GldE	*F. lacisediminis*	WP_264368811
Secretion system T9SS	type IX secretion system outer membrane channel protein PorV	*F. cucumis*	WP_073583464
Secretion system T9SS	cell surface protein SprA	*F. celericrescens*	WP_166235604
Secretion system T9SS	porin family protein	*F. celericrescens*	WP_166236975
Secretion system T9SS	PorV/PorQ family protein	*F. channae*	WP_231836252
Secretion system T9SS	type IX secretion system sortase PorU	*F. celericrescens*	WP_166237543
hemin transport	hemophore HmuY	*F. profundi*	WP_140998428
iron transport	metal ABC transporter ATP-binding protein	*F. tegetincola*	WP_035759166
iron transport	CcoQ/FixQ family Cbb3-type cytochrome c oxidase assembly chaperone	*F. channae*	WP_231834627
iron transport	Helical backbone metal receptor TroA-like domain	*F. tegetincola*	WP_026977525
iron transport	ferrous iron transport protein B	*F. cheniae*	WP_133608873
iron transport	ferrous iron transport protein A	*F. sasangense*	WP_026725509
iron transport	iron-sulfur cluster assembly accessory protein	*F. celericrescens*	WP_166235486
iron transport	Fe-S cluster assembly protein SufB	*F. sasangense*	WP_026725389
iron transport	Fe-S cluster assembly ATPase SufC	*F. tibetense*	WP_113988376
iron transport	Fe-S cluster assembly protein SufD	*F. saliperosum*	WP_023576282
iron transport	cysteine desulfurase SufS	*F. sasangense*	WP_026725384

iron transport	SufE family protein	*F. sasangense*	WP_026725383
siderophore transport	Iron(III) dicitrate transport protein FecA	*F. limnosediminis*	ESU27594
siderophore transport	MotA/TolQ/ExbB proton channel family protein	*F. cyclinae*	WP_231842879
siderophore transport	Biopolymer transport protein ExbD	*F. channae*	WP_231835470
siderophore transport	ATP-binding cassette domain-containing protein	*F. lacisediminis*	WP_264369289
siderophore transport	ABC transporter permease	*F. cheniae*	WP_133606348
siderophore transport	TonB-dependent receptor	*F. profundi*	WP_140998429
siderophore transport	energy transducer TonB	*F. cucumis*	WP_073580915
transcriptional regulation	Ferric uptake regulator Fur	*F. celericrescens*	WP_166235861
transcriptional regulation	metal-dependent transcriptional regulator	*F. proteolyticum*	WP_194093556
transcriptional regulation	AraC family transcriptional regulator	*F. proteolyticum*	WP_194093574
transcriptional regulation	sigma-70 family RNA polymerase sigma factor	*F. celericrescens*	WP_166235354
iron storage	ferritin	*F. tibetense*	WP_113987678
iron storage	DNA starvation/stationary phase protection protein	*F. flevense*	WP_073242182
	Hemolysins proteins containing CBS domains	*F. proteolyticum*	WP_194095253

Table S2. Virulence-associated features of strain LB-N7^T obtained with VFDB database.

VF class	Virulence factor	Strand	Rast annotation	E-value	% Identity	% Coverage	Description
Adherence	GroEL	-	Heat shock protein 60 kDa family chaperone GroEL	0	62.98	96.00	WP_011967678, chaperonin GroEL [*Clostridium*]
Adherence	LPS O-antigen	-	UDP-glucose 4-epimerase	6.00E-133	60.8	94.00	WP_003163315, NAD-dependent epimerase/dehydratase family protein [*Pseudomonas*]
Antiphagocytosis	Capsular polysaccharide	+	dTDP-4-dehydrorhamnose 3,5-epimerase	1.00E-67	51.34	99.00	WP_011261024, dTDP-4-dehydrorhamnose 3,5-epimerase [*Aliivibrio fischeri*]
Antiphagocytosis	Capsular polysaccharide	-	UDP-N-acetylglucosamine 2-epimerase	7.00E-169	62.23	96.00	WP_011149314, UDP-N-acetylglucosamine 2-epimerase (non-hydrolyzing) [*Vibrio vulnificus]* fue suprimida ya que no había sido anotada en otro genoma
Iron uptake	Heme biosynthesis	+	Glutamate-1-semialdehyde 2,1-aminomutase	5.00E-168	54.05	96	WP_011609384, glutamate-1-semialdehyde 2,1-aminomutase [*Histophilus somni*]
Other	O-antigen	-	GDP-L-fucose synthetase	1.00E-129	55.20	95.00	WP_002223297, GDP-L-fucose synthase [*Yersinia pseudotuberculosis* complex]
Other	O-antigen	-	GDP-mannose 4,6-dehydratase	4.00E-172	64.66	96.00	WP_011191888, GDP-mannose 4,6-dehydratase [*Yersinia pseudotuberculosis* complex]
Secretion system	T4SS effectors	-	Enoyl-[acyl-carrier-protein] reductase [NADH]	1.00E-179	61.81	98.00	WP_005771488, CBU_0270 family Dot/Icm type IV secretion system effector [*Coxiella burnetii*]
Secretion system	T6SS-II	+	Chaperone protein ClpB (ATP-dependent unfoldase)	0	55.34	89	WP_014226431, ATP-dependent chaperone ClpB [*Klebsiella*]

Toxin	Cytolysin	-	Transcriptional regulator, Xre family	4.00E-24	55.56	95	WP_002370931, cytolysin regulator CylR2 [*Enterococcus*]
Enzyme	Streptococcal enolase	-	Enolase	0	65.81	98	WP_002897814, surface-displayed alpha-enolase [*Streptococcus sanguinis*]
Immune evasion	Polysaccharide capsule	+	UDP-glucose 4-epimerase	7.00E-118	50.59	98	WP_000996578, UDP-glucose 4-epimerase GalE [*Bacillus cereus* group]
Stress adaptation	Catalase-peroxidase	-	Catalase-peroxidase KatG	0	58.22	94	WP_011560076, catalase/peroxidase HPI [*Mycolicibacterium monacense*]
